# Stem Cell Ophthalmology Treatment Study (SCOTS): Bone Marrow-Derived Stem Cells in the Treatment of Stargardt Disease

**DOI:** 10.3390/medicines8020010

**Published:** 2021-02-03

**Authors:** Jeffrey N. Weiss, Steven Levy

**Affiliations:** 1 Coral Ridge Medical Center, 821 Coral Ridge Drive, Coral Springs, FL 33065, USA; jweissmd@me.com; 2 MD Stem Cells, 3 Sylvan Road South, Westport, CT 06880, USA

**Keywords:** Stargardt Disease, macular degeneration, SGDT1, bone marrow-derived stem cells, BMSC, stem cells, hereditary maculopathy, juvenile macular degeneration, hereditary macular degeneration, ABCA4 mutation

## Abstract

**Background:** Stargardt Disease is the most common inherited macular degeneration, typically resulting in progressive central vision loss and legal blindness at an early age. We report regarding 34 eyes with Stargardt Disease treated in the Stem Cell Ophthalmology Treatment Study (SCOTS and SCOTS2). **Methods:** Autologous bone marrow was processed, separating the stem cell fraction which was provided Arms using retrobulbar, subtenons, intravitreal or subretinal and intravenous. The follow-up period was one year. **Results:** Of the 34 treated eyes, 21 (61.8%) improved, 8 (23.5%) remained stable, and 5 (14.7%) showed continued progression of their disease. Results were statistically significant with *p* = 0.0004. The average central vision improvement following treatment was 17.96% (95%CI, 16.39–19.53%) and ranged up to 80.5%. Of 17 patients treated, 13 (76.5%) showed visual acuity improvement in one or both eyes, 3 patients (17.6%) showed no net loss, and 1 worsened as a consequence of disease progression; 94.1% of patients had improved vision or remained stable. There were no adverse events. **Conclusions:** Patients with Stargardt Disease may potentially benefit from autologous bone marrow-derived stem cells (BMSC) as provided in SCOTS. Improvement or stabilization of vision was found to occur for the vast majority of reported patients and findings were highly statistically significant.

## 1. Introduction

Stargardt Disease (STGD) [1,2,3] is the most common form of inherited juvenile macular degeneration with a prevalence of 1 to 8000–10,000. There is an association with several different genes:

STGD1: the most common type (95% of cases), is autosomal recessive and caused by mutations in the ABCA4 gene, although it can also be associated with a mutation in CNGB3. Defective ABCA4 affects the ATP-binding cassette transporter protein causing the formation of toxic vitamin A biretinoids. Damaged retinal cells will form lipofuscin in the retinal pigment epithelium, the characteristic finding in this condition. There are over 1000 mutations of ABCA4 known to cause STGD1 and related retinal diseases.

STGD3: This is a rare dominant type of Stargardt Disease caused by a mutation in the ELOVL4 gene.

STGD4: This type is associated with mutations in PROM1.

The Stem Cell Ophthalmology Treatment Study (SCOTS) is a National Institutes of Health (NIH) registered study, NCT 01920867. It is being conducted under the supervision of an Institutional Review Board and may be accessed through www.clinicaltrials.gov. The study is using autologous (same person) stem cells extracted from the bone marrow (bone marrow-derived stem cells or BMSC) which are separated and used with local and systemic applications for diseases of the retina and optic nerve. This is the largest such study to date and has reported results in multiple peer-reviewed publications.

SCOTS is a non-randomized study conducted in an open label fashion to assess the benefit of BMSC in ophthalmic disease. The study uses historic control with all admitted patients receiving active stem cell treatment; there is no sham or placebo arm. Bone marrow aspirated from the posterior Iliac Crest is separated to provide Bone Marrow-Derived Stem Cells (BMSC) within the stem cell concentrate.

A description of the study from the records of the NIH [4] follows.

Inclusion Criteria:

- Have objective, documented damage to the retina or optic nerve unlikely to improve; OR
- Have objective, documented damage to the retina or optic nerve that is progressive;
- AND have less than or equal to 20/40 best-corrected central visual acuity in one or both eyes AND/OR an abnormal visual field in one or both eyes;
- Be at least 3 months post-surgical treatment intended to treat any ophthalmologic disease and stable;
- If under current medical therapy (pharmacologic treatment) for a retinal or optic nerve disease, be considered stable on that treatment and unlikely to have visual function improvement (for example, glaucoma with intraocular pressure stable on topical medications but visual field damage);
- Have the potential for improvement with BMSC treatment and be at minimal risk of any potential harm from the procedure;
- Be over the age of 18;
- Be medically stable and able to be medically cleared by their primary care physician or a licensed primary care practitioner for the procedure. Medical clearance means that in the estimation of the primary care practitioner, the patient can reasonably be expected to undergo the procedure without significant medical risk to health.

Exclusion Criteria:

- Patients who are not capable of an adequate ophthalmologic examination or evaluation to document the pathology;
- Patients who are not capable or not willing to undergo follow-up eye exams with the principle investigator or their ophthalmologist or optometrist as outlined in the protocol;
- Patients who are not capable of providing informed consent;
- Patients who may be at significant risk to general health or to the eyes and visual function should they undergo the procedure;

Intervention:

- Procedure: RB (Retrobulbar)
  Retrobulbar injection of Bone Marrow-Derived Stem Cells (BMSC)
  Other Name: Retrobulbar injection of stem cells
- Procedure: ST (Subtenon)
  Subtenon injection of Bone Marrow-Derived Stem Cells (BMSC)
  Other Name: Subtenon injection of stem cells
- Procedure: IV (Intravenous)
  Intravenous injection of Bone Marrow-Derived Stem Cells (BMSC)
  Other Name: Intravenous injection of stem cells
- Procedure: IVIT (Intravitreal)
  Intravitreal injection of Bone Marrow-Derived Stem Cells (BMSC)
  Other Name: Intravitreal injection of stem cells
- Procedure: IO (Intraocular)
  Intraocular injection of Bone Marrow-Derived Stem Cells (BMSC) with vitrectomy prior to intraocular injection. For example, may include a larger amount of stem cells in the intravitreal cavity, intraneuronal injections, or subretinal injections of stem cells.
  Other Name: Intraocular injection of stem cells with vitrectomy.
- Active Comparator: RB, ST, IV
  Injections of BMSC retrobulbar (RB), subtenon (ST) and intravenous (IV)
  Interventions:
  ○ Procedure: RB (Retrobulbar)
  ○ Procedure: ST (Subtenon)
  ○ Procedure: IV (Intravenous)
- Active Comparator: RB, ST, IV, IVIT
  Injections of BMSC retrobulbar, subtenon, intravenous and intravitreal (IVIT)
  Study Arms:
  ○ Procedure: RB (Retrobulbar)
  ○ Procedure: ST (Subtenon)
  ○ Procedure: IV (Intravenous)
  ○ Procedure: IVIT (Intravitreal)
- Active Comparator: RB, ST, IV, IO
  Injection of BMSC retrobulbar, subtenon, intravenous and intraocular (IO) with vitrectomy
  Interventions:
  ○ Procedure: RB (Retrobulbar)
  ○ Procedure: ST (Subtenon)
  ○ Procedure: IV (Intravenous)
  ○ Procedure: IO (Intraocular)

## 2. Materials and Methods

Patients were assessed using their most recent ophthalmologic exam from their own provider. In addition, admitted patients received a complete eye examination which included past ophthalmic and medical history, assessment of vision including best-corrected Early Treatment Diabetic Retinopathy Study (ETDRS) and Snellen, biomicroscopy of anterior structures, intraocular pressures, and careful examination of the fundus following dilation. Visual field examinations, photos of the fundus and OCT (ocular coherence tomography) were included. Suspected neovascularization was assessed with fluorescein angiography. Evaluation of visual loss was gained from historical information obtained through a review of medical records and patient-provided history.

To quantify differences in lines of vision, all acuity values were converted to the logarithm of the minimum angle of resolution (logMAR) scores, where each 0.1 logMAR unit represents 1 line of visual acuity (Table 1).

Careful informed consent with discussion reviewing options and emphasizing the experimental nature of the procedure was obtained. Surgeries were provided by an author (J.N.W.) and were done as out-patient procedures at an ambulatory surgery center.

A preoperative eye exam was done the day prior to the procedure and postoperatively by the surgeon (JNW). Follow-up eye exams were at one, three, six, and 12 months following the procedure and could be conducted by the patient’s own eye physician to avoid the burden of repeated travel.

The Institutional Review Board provided ethics supervision; approval code 2013-0019 approved on 30 July 2013.

## 3. Results

As each eye met eligibility criteria, both eyes of 17 patients were treated for a total of 34 eyes (Table 2). Change in lines of vision is noted in Table 3. There were no intraoperative or postoperative surgical complications.

The preoperative visual acuity in the 34 eyes treated ranged from CF 6′ to 20/40 + 2. Logarithm of the minimum angle of resolution or LogMAR measurement of vision in these eyes ranged from 1.85 to 0.28. Calculation of vision change following treatment was determined by dividing the difference in logMAR acuity from pre to post treatment (delta logMAR) by the pre-treatment acuity. The mean preoperative vision in LogMAR across all 34 eyes was 1.30; variance was 0.3493 and standard deviation was 0.591.

Of the 34 treated eyes, 21 (61.8%) improved, 8 (23.5%) remained stable, and 5 (14.7%) showed progression of their disease. The mean visual acuity change across all eyes following treatment in SCOTS was an increase of +0.2344 logMAR giving an average improvement of 17.96% (95% CI, 16.39–19.53%) or 1.59 lines of vision. In the 21 eyes that improved, the mean preoperative vision in LogMAR was 1.23; mean logMAR improvement was +0.4095 giving an average visual acuity improvement of 33.3% or 4.29 lines of vision. Of the 34 treated eyes, 85.3% improved or remained stable in the follow-up period.

Using a 2-tail Students *t*-test evaluation of LogMAR vision results, comparing the data set of LogMAR change to µ = 0, the improvement following treatment of Stargardt in the SCOTS study was highly statistically significant with *p* = 0.0004.

Assessing overall patient response to treatment during the follow-up period, 13 patients (76.5%) showed improvement in one or both eyes, 3 patients (17.6%) showed no net loss, and 1 worsened as a consequence of disease progression. In terms of patient benefit, 94.1% of patients improved or remained stable during the follow-up period. This contrasts with untreated Stargardt Disease patients who experience an average acuity loss of between 0.054 and 0.114 logMAR per year [5].

Any decrease in acuity was noted after the postoperative period and was not a result of any adverse event, but rather the natural progression of the disease. In the 5 eyes that showed progression from Stargardt Disease, mean preoperative vision in logMAR was 1.59 with an average postoperative change in logMAR of −0.144. This was in keeping with prior research showing between 0.054 and 0.114 logMAR/year expected loss of acuity in the ProgStar study of Stargardt [5]. Of note, only 1 of 17 patients had continued disease progression in both eyes; in other patients, the fellow eye either improved or was stable.

The presence/absence of a posterior vitreous detachment (PVD) did not appear significant. There did not appear to be a significant difference in the effectiveness of the Arm 1 versus the Arm 2 or Arm 3 procedures. Stem cell concentrate placed subretinal within large atrophic areas at the posterior pole resolved over several months. The same finding was observed in Arm 3 patients undergoing the SCOTS procedure for age-related macular degeneration [6].

## 4. Discussion

Efforts to address the lack of therapy for Stargardt Disease have included oral therapy, intravitreal injections. stem cells, and gene therapy.

ALK-001 is a synthetic vitamin A once-a-day pill that prevents the formation of toxic vitamin A dimers in the eye. This form of vitamin A is not readily converted to lipofuscin, slowing their deposition and potentially slowing vision loss. It is being testing in a Phase II multicenter clinical trial (ClinicalTrials.gov Identifier NCT02402660) and other vitamin A variants have been explored [7,8]. However, oral intake of excessive vitamin A has been shown to increase lipofuscin deposition in animal models, possibly worsening vision loss in Stargardt.

Ocata therapeutics (previously Advanced Cell Technology) has completed a Phase I/IIa multicenter trial using retinal pigment epithelial cells derived from human embryonic stem cells [9]. No ocular safety issues were encountered, though, side effects from patient immunosuppression were observed. This company was subsequently acquired by Astelas Pharma and follow-up studies without treatment were reported to have continued.

The purpose of gene replacement therapy is to attempt to decrease or stop additional retinal tissue loss by targeting photoreceptors [10,11,12,13]. Though the most experience had been obtained with Adeno-associated virus vectors, the ABCA4 gene is larger than the capacity of the current AAV vector, which makes the lentivirus the vector of choice. A lentivirus vector has been used in a Phase I/II clinical trial (ClinicalTrials.gov Identifier: NCT01367444), and no safety concerns were identified [14].

The multicenter Natural History of the Progression of Atrophy Secondary to Stargardt Disease [15] studies are describing the natural history of disease progression of the condition. They determined that the rate of progression was mainly determined by the initial lesion size.

Other studies have supported the efficacy of stem cells in the treatment of AMD and Stargardt Disease [16]. Our group has previously demonstrated benefit of the SCOTS BMSC treatment in several genetic eye conditions improving visual acuity.

Patients with Leber Hereditary Optic Neuropathy experienced visual acuity benefit that was stable over the one-year follow-up period. Those results have been determined to be statistically significant [17]. Additional reports on other hereditary conditions including Retinitis Pigmentosa [18], Ushers Syndrome [19], and Autosomal Dominant Optic Atrophy [20] have also shown statistically significant visual acuity improvements.

The explanation for using stem cells to treat patients with visual acuity loss of genetic etiology is that the bone marrow-derived stem cell fraction may benefit damaged but repairable cells via neuroprotection mechanisms, reduce ongoing immunogenic damage, transfer cytoplasmic structures including mitochondria and lysosomes to damaged cells, and produce neuronal transformation of BMSC which can fuse with Müller cells to then transdifferentiate into specialized neurons including ganglion and amacrine neurons [21].

Although the etiology of genetically acquired vision loss varies, there may be common and accessible repair mechanisms using BMSC. Mechanisms of action for BMSC have been presented and discussed in prior papers reporting the results of SCOTS. The BMSC fraction includes mesenchymal stem cells, other stem cells, as well as platelets. These produce and release exosomes which contain Nerve Growth Factor, various Neurotrophic Factors, and miRNA (messenger interference Ribonucleic Acid). BMSC have been shown to transfer cytoplasmic structures including mitochondria and lysosomes to damaged cells, and to provide immune modulation.

The most common mutations causing Stargardt Disease affect the ABCA4 (ATP Binding Cassette subfamily A member 4) gene.

Various mutations of this gene are also associated with Retinitis Pigmentosa and Cone-Rod Dystrophy, as well as an increased patient risk of AMD.

The ABCA4 transporter is located primarily in the retina and is one of multiple ABCA proteins associated with lipid transport across cell membranes. It is responsible for transporting N-retinylidene-PE from the lumen to the cytoplasmic side of the disc membrane, allowing conversion of all-trans retinal to all-trans retinol, which is then transported into the RPE (Retinal Pigment Epithelial) cells. There it converts to 11-cis retinal, which is transported back into the outer segment of the photoreceptor to combine with opsin and regenerate rhodopsin or cone opsin completing the visual cycle.

With an abnormal ABCA4 transporter, removal of the N-retinylidene-PE is impaired which allows it to react with all-trans retinal to form a diretinal derivative called A2PE. Because outer segments of the photoreceptors are constantly renewed, RPE cells ingest the A2PE in phagosomes which fuse with lysosomes to degrade. However, A2PE can only be hydrolyzed to N-retinylidene-N-retinyl-ethanolamine (A2E) and cannot be further broken down. A2E accumulates progressively in the RPE cells as a component of lipofuscin. Lipofuscin is a complex combination of oxidized macromolecules which can accumulate in different tissues. With blue light exposure, lipofuscin in the RPE can form epoxides which can cause RPE apoptosis. Ultimately, RPE cell death causes photoreceptor cell death and decreased vision.

While speculative, it is useful to consider how the treatment with BMSC may be affecting Stargardt Disease on the cellular and molecular level. Different mutations of ABCA4 cause the protein to behave differently in terms of its ability to bind N-retinylidene-PE substrate in the absence of ATP [22]. It is conceivable that the transfer of mitochondria by BMSC to photoreceptors may improve mutated ABCA4 substrate binding by increasing the presence of ATP and ultimately reducing A2E levels. Mitochondrial dysfunction and mitochondrial DNA damage may result in incomplete mitophagy and increase lipofuscinogenesis. Mitochondria transferred by BMSC could help mitigate these processes. Cytoplasmic transfer of functional lysosomes by BMSC to distressed RPE cells may be occurring. This could potentially facilitate the normal degradation of cellular proteins, otherwise stymied by ineffective processing of A2E, thereby improving RPE function. Chronic inflammation plays a role in RPE damage [8], and therefore the immune modulation provided by BMSC may also be a factor in clinical improvement.

Stargardt Disease patients with preoperative 20/400 or worse vision did not generally improve beyond 20/100. While such degrees of visual recovery are meaningful and beneficial from a functional perspective, it suggests that recovery of full macular function with a single treatment may be difficult. There are exceptions, as evidenced by Patient #1 who went from Counting Fingers to 20/50 + 2.

Patients with macular function, with a visual acuity better than 20/200, generally maintained macular function, and in some cases experienced significant improvement in visual acuity. This may suggest that earlier treatment could provide greater opportunity for the mechanisms of action of the BMSC to reverse damage and offer neuroprotection. Stargardt Disease can result from many hundreds of different mutations in ABCA4 or other genes. There may be phenotype contributions from other genes compensating or exacerbating the deficits of the abnormal ATP Binding Cassette protein. Therefore, larger cohorts with careful genetic mapping would be useful to help determine, on a personalized basis, the degree of benefit potentially expected.

It is a limitation of this study that most of the treated patients had not undergone genetic testing. Another study limitation was that, after the immediate postoperative period, examinations were performed by the patient’s local physician, who did not use ETDRS visual acuities. All the patients came from a distance and they returned to their local physician for follow-up. Having the postoperative examinations performed by a physician, unassociated with SCOTS, was felt to eliminate any potential bias. Although preoperative ETDRS visual acuities were taken preoperatively, they were subsequently excluded since there were no postoperative comparisons.

## 5. Conclusions

The use of BMSC in the method stipulated in the Stem Cell Ophthalmology Treatment Study (now SCOTS2) has been shown to provide statistically significant (*p* = 0.0004) visual benefit in patients with Stargardt Disease. Improvements in visual acuity of up to 80.5% and averaging 17.96% (95% CI, 16.39–19.53%) were noted. Patients benefitted with 94.1% improving or remained stable during the follow-up period. While further investigation is needed to determine which specific mutations and degrees of maculopathy will be most likely to benefit, the SCOTS2 BMSC approach may be considered a treatment option for patients with this condition.

## Figures and Tables

**Table 1:** Assessment of lines of vision improvement. Equivalent decimal, Snellen and LogMAR acuity measurements.

Decimal	Snellen	LogMAR			
0.800	20/25	0.1			
0.625	20/32	0.2			
0.500	20/40	0.3			
0.400	20/50	0.4			
0.317	20/63	0.5			
0.250	20/80	0.6			
0.200	20/100	0.7			
0.160	20/125	0.8			
0.125	20/160	0.9			
0.100	20/200	1			
0.080	20/250	1.1			
0.063	20/320	1.2			
0.050	20/400	1.3			
0.040	20/500	1.4			
0.033	20/600	1.5			
0.025	20/800 CF 10 ft.	1.6			
0.020	20/1000 CF 8 ft.	1.7			
0.017	20/1200 ~CF 7 ft.	1.8	0.014	20/1400 CF 6 ft.	1.85
0.013	20/1600 CF 5 ft.	1.9	0.011	20/1800 CF 4 ft.	1.95
0.010	20/2000 CF 2 ft.	2			
		2.1			
		2.2			
		2.3			
		2.4			
		2.5			
		2.6			
		2.7			
		2.8			
		2.9			
0.001	20/20,000 HM	3			

**Table 2:** A summary of each patient’s demographic data, Snellen Acuity pre and post treatment, LogMAR pre and post treatment, percent vision change, and bilateral vision results.

Patient No.	Age (yrs) Gender	Medical History	Ocular History	Family History	PVD OD/OS	Arm #	Pre-Va OD	Post-Va OD	Pre-Va OS	Post-Va OS	Comments	Pre-Va LogMAR OD	Post-Va LogMAR OD	Pre-Va LogMAR OS	Post-Va LogMAROS	Delta LogMAR % Change OD	Delta LogMAR % Change OS	Bilateral Vision
1	71/M	DM/S/P prostate surgery(CA)	gene testing confirmed Stargardt	Glaucoma/DM/CA	Y/Y	2/2	CF 6′	CF (?)	CF 6′	20/50 + 2		1.85	1.85?	1.85	0.36	0 0%	+1.49 80.5%	improved
2	36/F	-	- gene testing	AMD	N/N	2/2	20/400	20/400	20/400	20/200 + 1		1.3	1.3	1.3	0.98	0 0%	+0.32 24.6%	improved
3	41/F	Hyperthryoidism	- gene testing	AMD/CA/DM	N/N	2/2	20/400	20/400	CF6′	CF2′		1.3	1.3	1.85	2	0 0%	(−0.15) (−10.3%)	no net loss
4	54/F	Cardiac valve abnormality/Asthma	- gene testing	AMD	Y/Y	2/2	Cf 6′	20/400	20/400	20/400	Significant VF improvement OD/OS	1.85	1.3	1.3	1.3	+0.55 29.7%	0 0%	improved
5	30/M	-	- gene testing	DM	N/N	2/2	20/50 − 1	20/50 + 1	20/40 + 2	20/40 + 2		0.41	0.38	0.28	0.28	+0.03 7.3%	0 0%	improved
6	53/M	Atrial Fib.	- gene testing	Stargardts/AMD	N/N	2/3	20/400	20/100	20/400	20/100		1.3	0.7	1.3	0.7	+0.6 46.2%	+0.6 46.2%	improved
7	51/M (brother of #6)	Hypertension	- gene testing	Stargardts/AMD	NN	2/2	20/200	20/70	20/100 + 1	20/60		1	0.55	0.69	0.49	+0.45 45%	+0.2 29%	improved
8	70/M	Hypertension/MI/S/P CABG/	gene testing confirmed Stargardts	AMD	Y/Y	1/2	20/400	20/200	CF 6′	20/250		1.3	1	1.85	1.1	+0.3 23%	+0.75 40.5%	improved
9	56/M	Hypertension	- gene testing	AMD	Y/Y	1/2	20/200 − 1	20/200 − 1	20/400	20/200 − 1		1	1	1.3	1	0	+0.3 23%	improved
10	30/M	-	- gene testing	Glaucoma	N/N	1/1	20/400	20/200	20/200	20/400		1.3	1	1	1.3	+0.3 23%	(−0.3) (−30%)	no net change
11	54/M	-	gene testing confirmed Stargardts	Stargardts	N/N	1/2	20/80 + 1	20/60 + 1	20/200	20/200 + 1		0.58	0.47	1	0.98	+0.11 19%	+0.02 2%	improved
12	28/M	-	- gene testing	-	N/N	2/3	20/200	20/150 − 1	20/200	20/150 − 1	1 ppd x12yrs	1	0.86	1	0.86	+0.14 14%	+0.14 14%	improved
13	65/F	-	- gene testing	Stargardts	N/N	2/3	20/400	20/400	20/400	20/400		1.3	1.3	1.3	1.3	0 0%	0 0%	no change
14	72/F	COPD	- gene testing	-	Y/N	3/2	CF5′	CF2′	CF5′	CF3′	cataracts OU	1.9	2	1.9	1.97	(−0.1) (−5.3%)	(−0.07) (−3.7%)	loss
15	26/M	-	- gene testing	-	N/N	2/2	20/400	20/200	20/400	20/200 − 1		1.3	1	1.3	1	+0.3 23%	+0.3 23%	improved
16	29/M	-	- gene testing	AMD/Glaucoma/DM	N/N	2/2	CF 6′	20/400 + 1	CF 6′	20/200 − 1		1.85	1.3	1.85	1	+0.55 29.7%	+0.85 45.9%	improved
17	53/M	Parkinsons/Cardiac valve abnormality	- gene testing	Stargardts	Y/Y	2/2	20/400	20/500	20/400	20/200		1.3	1.4	1.3	1	(−0.1) (−7.7%)	+0.3 23%	improved
													34 eyes	avg preop	=1.30	avg delta +0.2344	change + 17.96%	
																*p* = 0.0004		
													21 eyes improved	improved		change +33.3%		

**Table 3:** Lines of vision results.

Patient No.	Postop Va Change OD	Postop Va Change OS	Postop Total Change in Best Va OU
1	No Change	>+12 lines	>+12 lines
2	No Change	>+3 lines	>+3 lines
3	No Change	−4 lines	No Change
4	>5 lines	No Change	>+5 lines
5	+/− No Change	No Change	+/− No Change
6	>+5 lines	>+5 lines	>+5 lines
7	>+3 lines	<+2 lines	<+2 lines
8	+3 lines	>+8 lines	No Change
9	No Change	No Change	No Change
10	+3 lines	−3 lines	No Change
11	+1 line	No Change	+1 line
12	+1 line	−1 line	No Change
13	No Change	No Change	No Change
14	−3 lines	−2 lines	−2 lines
15	+3 lines	+3 lines	+3 lines
16	+5 lines	+8 lines	+8 lines
17	−1 line	+3 lines	+3 lines

## Data Availability

Data is contained within the article.
